# Spatial distribution of D1R- and D2R-expressing medium-sized spiny neurons differs along the rostro-caudal axis of the mouse dorsal striatum

**DOI:** 10.3389/fncir.2013.00124

**Published:** 2013-07-29

**Authors:** Giuseppe Gangarossa, Julie Espallergues, Philippe Mailly, Dimitri De Bundel, Alban de Kerchove d'Exaerde, Denis Hervé, Jean-Antoine Girault, Emmanuel Valjent, Patrik Krieger

**Affiliations:** ^1^CNRS, UMR 5203, Institut de Génomique FonctionnelleMontpellier, France; ^2^INSERM, U661Montpellier, France; ^3^Universités de Montpellier 1 & 2, UMR 5203Montpellier, France; ^4^CNRS UMR 7224, INSERM UMRS 952, Université Pierre et Marie CurieParis, France; ^5^Laboratory of Neurophysiology, ULB Neuroscience Institute, Université Libre de BruxellesBrussels, Belgium; ^6^INSERM, UMRS 839Paris, France; ^7^UMRS 839, Université Pierre et Marie Curie-Paris 6Paris, France; ^8^Institut du Fer à MoulinParis, France; ^9^Department of Neuroscience, Karolinska InstitutetStockholm, Sweden

**Keywords:** medium-sized spiny neurons, BAC transgenic mice, dopamine D1 and D2 receptors, adenosine A2a receptor

## Abstract

The striatum projection neurons are striatonigral and striatopallidal medium-sized spiny neurons (MSNs) that preferentially express D1 (D1R) and D2 (D2R) dopamine receptors, respectively. It is generally assumed that these neurons are physically intermingled, without cytoarchitectural organization although this has not been tested. To address this question we used BAC transgenic mice expressing enhanced green fluorescence (EGFP) under the control of *Drd1a* or *Drd2* promoter and spatial point pattern statistics. We demonstrate that D1R- and D2R-expressing MSNs are randomly distributed in most of the dorsal striatum, whereas a specific region in the caudal striatum, adjacent to the GPe, lacks neurons expressing markers for indirect pathway neurons. This area comprises almost exclusively D1R-expressing MSNs. These neurons receive excitatory inputs from the primary auditory cortex and the medial geniculate thalamic nucleus and a rich dopamine innervation. This area contains cholinergic and GABAergic interneurons but apparently no D2R/A2aR modulation because no fluorescence was detected in the neuropil of *Drd2-EGFP* or *Drd2-Cre*, and *Adora-Cre* BAC transgenic mice crossed with reporter mice. This striatal area that expresses calbindin D28k, VGluT1 and 2, is poor in μ opiate receptors and preproenkephalin. Altogether, the differences observed in D1R-MSNs, D2R-MSNs, and interneurons densities, as well as the anatomical segregation of D1R- and D2R/A2aR-expressing MSNs suggest that there are regional differences in the organization of the striatum.

## Introduction

The striatum is the main input structure of the basal ganglia, an ensemble of integrative subcortical nuclei enabling the elaboration of complex motor behavior (Bolam et al., 2000; Graybiel, 2004; Nicola, 2007; Gerfen and Surmeier, 2011). The GABAergic medium-sized spiny projections neurons (MSNs) that constitute the vast majority of striatal neurons (~95% in rodents) integrate cortical and thalamic excitatory inputs and are modulated by dopaminergic inputs and striatal interneurons (Gerfen and Surmeier, 2011). Two major subpopulations of MSNs have been defined on the basis of their projection targets (Gerfen and Surmeier, 2011). Thus, MSNs projecting to the external globus pallidus (GPe) participate in the indirect striatopallidal pathway while MSNs innervating the internal globus pallidus (GPi), or entopeduncular nucleus, and the substantia nigra pars reticulata (SNr) form the direct striatonigral pathway (Gerfen and Young, 1988; Gerfen et al., 1990; Gerfen and Surmeier, 2011).

In addition to their participation in different anatomical pathways, the two MSNs populations display distinct and specific molecular profiles (Gerfen et al., 1990; Valjent et al., 2009). Among them, striatopallidal MSNs are enriched in enkephalin, D2 dopamine (D2R) and A2a adenosine (A2aR) receptors, whereas striatonigral MSNs express substance P, dynorphin and D1 dopamine receptor (D1R) (Gerfen and Surmeier, 2011). Although the degree of segregation of the two populations of MSNs has been continuously debated, recent anatomical and functional observations obtained in BAC transgenic mice confirmed the simple model of striatal output organization initially proposed (Valjent et al., 2009; Bertran-Gonzalez et al., 2010) and its functional correlates (Durieux et al., 2009, 2011; Kravitz et al., 2010).

In contrast to layered brain structures such as the cerebral cortex or the hippocampus, the striatum lacks easily identified cytoarchitecture (Goldman-Rakic, 1982; Hardman et al., 2002). Although functional striatal territories have been defined based on the arrangement of the corticostriatal and thalamostriatal inputs and on the patch/matrix compartmentalization (Graybiel, 1984; Selemon and Goldman-Rakic, 1985; Haber et al., 2006), the current view of the anatomical organization of the striatum is that D1R- and D2R-containing MSNs are intermingled throughout the striatum, displaying a uniform organization (Lanca et al., 1986; Gerfen, 1989). However, the spatial distribution of D1R- and D2R-expressing MSNs has never been precisely studied. Here, using a variety of BAC transgenic mice, we show that the distribution of the two populations varies along the rostro-caudal axis of the dorsal striatum. Indeed, we identify a specific region in the caudal striatum, adjacent to the GPe, which appears to lack neurons expressing markers for indirect pathway neurons. Altogether, our study suggests the intriguing possibility of the existence of a region of the mouse striatum where the information processing could be based principally on D1R-expressing MSNs.

## Materials and methods

### Mouse mutants

Male, 8–10-week old, *Drd2-EGFP* (*n* = 20 Swiss-Webster and 6 C57BL/6N background, founder *S118*), *Drd1a-EGFP* (*n* = 4 Swiss-Webster and *n* = 4 C57BL/6N background, founder *X60*), *Drd2-Cre* (*n* = 8 C57BL/6J background, founder *ER44*), *Adora2a-Cre* (*n* = 5 C57BL/6J background) hemizygous mice were used in this study. BAC *Drd2*-, *Drd1a-EGFP* and *Drd2-Cre* mice were generated by GENSAT (Gene Expression Nervous System Atlas) at the Rockefeller University (New York, NY) (Gong et al., 2007) and BAC *Adora2a-Cre* were generated at Laboratory of Neurophysiology, ULB (Brussels) (Durieux et al., 2009). *NLS-LacZ-Tau^mGFP^:loxP* (kindly provided by Dr Silvia Arber) (Hippenmeyer et al., 2005), *Rosa26:loxP* (Srinivas et al., 2001) and ***R****26R* ***C****AG-boosted* ***E****GFP:LoxP* (*RCE:LoxP)* (kindly provided by Dr Gord Fishell) (Miyoshi et al., 2010) mice were used as reporter to compare the patterns of expression in different mouse lines. Five males C57BL/6 (8–10 weeks) (Charles River, France) and 9 males *Drd2-EGFP* were used for tract-tracing experiments. Mice were maintained in a 12 h light/dark cycle, in stable conditions of temperature and humidity, with food and water *ad libitum*. All experiments were in accordance with the guidelines of the French Agriculture and Forestry Ministry for handling animals (authorization number/license D34-172-13).

### Tract-tracing studies

Surgeries were performed on 8-weeks old C57BL/6J or *Drd2-EGFP* mice. Animals were anesthetized with a mixture of ketamine (Imalgene 500, 50mg/ml, Merial), 0.9% NaCl solution (weight/vol) and xylazine (Rompun 2%, 20 mg/ml, Bayer) (2:2:1, i.p., 0.1 ml/30 g) and mounted on a stereotaxic apparatus. The microinjection needle was connected to a 10 μ l Hamilton syringe and filled with adeno-associated virus (AAV) containing ChR2-mCherry (AAV_2/1_.CAG:ChR2.mCherry, UPenn vector core, Philadelphia, USA) used as an anterograde tracer or cholera toxin subunit B fluorescent (CTB, Alexa Fluor 594 conjugate, Molecular Probes, Leiden, The Netherlands) used as a retrograde tracer. Microinjection needle was placed into the SN (*A*/*P* = −3.0 mm; Lat. = +1.55 mm; *D*/*V* = −4.25 mm) or VTA (*A*/*P* = −2.9 mm; Lat. = +0.4 mm; *D*/*V* = −4.5 mm) and 0.2 μ l was injected over 5 min. The injector was left in place for an additional 5 min to allow for diffusion of virus or toxin particles away from injection site. Wounds of mice were sealed by suture. Animals were then returned to their home cages for a 14 days survival period.

### 6-OHDA lesion

*Drd2-EGFP* mice were anaesthetized with a mixture of ketamine (Imalgene 500, 50 mg/ml, Merial), 0.9% NaCl (weight/vol) solution and xylazine (Rompun 2%, 20 mg/ml, Bayer) (2:2:1, i.p., 0.1 ml/30 g) and mounted on a stereotaxic apparatus. The surface of cranium was exposed and a hole was drilled at the appropriate coordinates. A cannula connected to a Hamilton 0.5 μl microsyringe was stereotaxically lowered to the SNc. The following coordinates were used: *AP* = −2.92 mm, *L* = −1.35 mm and *V* = −4.25 mm (Franklin and Paxinos, 2007). A volume of 0.2 μl of 6-OHDA^*^HCl (3 μg/μl of free base, dissolved in ascorbic acid 0.02%) was unilaterally injected at a rate of 0.05 μl/min. The intra SNc microinjection of 6-OHDA was preceded by the administration of desipramine (20 mg/kg, i.p.), a selective inhibitor of norepinephrine reuptake (Frazer, 2000), in order to protect noradrenergic fibers (Fulceri et al., 2006). Following the injections the cannula was left in place for another 4 min before retraction. Mice were allowed to recover for a period of 2 weeks before experiments.

### Ibotenic acid lesions

The same procedure described above was used for ibotenic acid lesions except that the cannula was lowered to the primary auditory cortex (Au1) or to the medial geniculate thalamic nucleus (MGV and MGM). The coordinates for Au1 and MGV injection sites were: *AP* = −2.54 mm, *L* = −4.1 mm, *V* = −2.3 mm and *AP* = −3.08 mm, *L* = −2.0 mm, *V* = −3.1 mm, respectively (Franklin and Paxinos, 2007). A volume of 0.2 μl of ibotenic acid (10 mg/ml, dissolved in PBS pH = 7.4) was unilaterally injected at a rate of 0.05 μl/min. Following the injection the cannula was left in place for another 4 min before retraction. Again mice were allowed to recover for a period of 2 weeks before experiments.

### Tissue preparation and immunofluorescence

Mice were rapidly anaesthetized with pentobarbital (500 mg/kg, i.p., Sanofi-Aventis, France) and transcardially perfused with 4% (weight/vol.) paraformaldehyde in 0.1 M sodium phosphate buffer (pH 7.5) (Bertran-Gonzalez et al., 2008). Brains were post-fixed overnight in the same solution and stored at 4°C. Thirty micrometer thick sections were cut with a vibratome (Leica, France) and stored at −20°C in a solution containing 30% (vol/vol) ethylene glycol, 30% (vol/vol) glycerol, and 0.1 M sodium phosphate buffer, until they were processed for immunofluorescence. Striatal areas were identified using a mouse brain atlas (Franklin and Paxinos, 2007): rostral level corresponds to approximately +1.18 mm relative to bregma and the caudal level to −1.85 mm. Sections were processed as follows: free-floating sections were rinsed in Tris-buffered saline (TBS: 0.25 M Tris and 0.5 M NaCl, pH 7.5), incubated for 5 min in TBS containing 3% H_2_O_2_ and 10% methanol (vol/vol), and then rinsed three times 10 min in TBS. After 15 min incubation in 0.2% (vol/vol) Triton X-100 in TBS, sections were rinsed three times in TBS again. Sections were then incubated for 1 h in a solution of BSA 3% in TBS. Finally, they were incubated overnight or 72 h at 4°C with the primary antibodies: chicken and rabbit anti-GFP (1:500 and 1:1000 respectively, Invitrogen), rabbit anti-vesicular glutamate transporter 1 (VGluT1) or anti-VGluT2 (1:1000 gift from S. El Mestikawy), mouse anti-tyrosine hydroxylase (TH) (1:1000, Millipore), rat anti-dopamine transporter (DAT) (1:1000, Millipore), mouse anti-NeuN (1:500, Millipore), mouse anti-D1R (1:500 gift from R. R. Luedtke), rabbit anti-Gα olf (1:500) (Hervé et al., 2001), rabbit anti-β-galactosidase (1:1000, Cappel, MP Biomedicals), guinea-pig anti-MOR (1:500 gift from T. Kaneko) mouse anti-DARPP-32 (1:1000 gift from P. Greengard), rabbit anti-calretinin (CalR), anti-calbindin-D28k and anti-parvalbumin (ParV) (1:1000, Swant), rabbit anti-neuropeptide Y (NPY) (1:400, Abcam), goat anti-ChAT (1:400, Millipore), rabbit anti-substance P (1:500, Millipore), rabbit anti-preproenkephalin (ppENK) (1:500, Neuromics) and rabbit anti-RFP (1:1000, MBL). For anti-ppEnk, anti-substance P (Sub P) and anti-MOR immunostainings, coronal sections were processed for antigen retrieval procedure with a 10 mM citrate buffer (0.05% Tween20, pH = 6) and heated at 75°C for 15 min. After incubation with the primary antibodies, sections were rinsed three times for 10 min in TBS and incubated for 45 min with goat or donkey Cy2-, Cy3- and Cy5-coupled (1:400, Jackson Lab) and/or goat A488 (1:400, Invitrogen). Sections were rinsed for 10 min twice in TBS and twice in TB (0.25 M Tris) before mounting in 1,4-diazabicyclo-[2.2.2]-octane (DABCO, Sigma-Aldrich).

Images covering the entire striatum used for 3D reconstruction were acquired using a Zeiss AxioImager Z1 and stitched together using the Carl Zeiss Panorama program. Confocal microscopy analysis was carried out at the Montpellier RIO Imaging Facility. Double- or triple-labeled images from each region of interest were obtained using sequential laser scanning confocal microscopy (Zeiss LSM510 META). Photomicrographs were obtained with the following band-pass and long-pass filter settings: GFP (band pass filter: 505–530), Cy3 (band pass filter: 560–615) and Cy5 (long-pass filter 650). The objectives and the pinhole setting (1 airy unit) remained unchanged during the acquisition of a series for all images. The thickness of the optical section is ~1.6 μm with a 20X objective and ~6 μm with a 10X objective. GFP labeled neurons were pseudo-colored green and other markers immunoreactive neurons were pseudo-colored magenta, red or blue. From the overlap of green and magenta, double-labeled neurons appeared white. The analysis was performed by counting nuclear GFP fluorescence (for assessment of D2R-positive cells) and Cy3-positive cells. Quantification of immunoreactive cells was performed using image analyzer software (Image-J). Striatopallidal neurons (D2R- and A2aR-expressing MSNs) and classes of interneurons (ChAT-, parvalbumin-, NPY- and calretinin-expressing interneurons) were quantified throughout the extension of the caudal striatal territory that lacks D2R/A2aR-containing MSNs either in *Drd2-EGFP* or *Adora2a-Cre* mice. Eighteen to twenty coronal slices per mouse covering the entire extension of this striatal territory (approximately from −1.46 to −2.06 mm relative to bregma) were stained with antibodies for GFP, ChAT, ParV, NPY, CalR and NeuN. Reported values (percentage and number of cells) represent the mean of two hemispheres.

### Images processing and 3D reconstruction model

All image processing, registration and 3D reconstruction were performed using IMOD package software, a set of open source image processing, modeling and display programs freely distributable developed at the Boulder Laboratory for 3-D Electron Microscopy of Cells (Kremer et al., 1996). IMOD package is freely available for Linux, Windows and Mac OS X systems, the different versions can be downloaded at http://bio3d.colorado.edu/imod/. The procedures to obtain 3D models have been described before (Mailly et al., 2010). Briefly, an image stack was built from stained Drd2-EGFP sections acquisitions. Sections were then aligned and on each section, contours of anatomical structures and Drd2-EGFP negative areas were manually delineated. For each delineated structure, an independent 3D object was calculated. Each resulting object could be displayed with different colors and rendered with specific light properties and could be rotated in any direction, allowing better visualization of internal structures. 3D model could be cut at defined *X*, *Y*, and *Z* planes by clipping planes into the 3D view.

### Spatial point pattern statistics

Spatial point patterns can be divided into three main categories of patterns (Diggle, 2003): aggregation (clustering), where events tend to attract other events; inhibition (dispersion), where events tend to repel other events and hence create a more regular pattern; and complete spatial randomness (CSR) where events are distributed randomly. The spatial distribution of cells was analyzed using Ripley's *K*-function as previously described (Eglen and Wong, 2008; Jafari-Mamaghani et al., 2010; Hansson et al., 2013). This method compares the experimentally measured density of cells surrounding each cell with the expected density if the distribution was following CSR (Ripley, 1988; Diggle, 2003). The estimated *K*-function value is abbreviated (^∧^*K*). The circumflex (^∧^) is used to denote that it is an estimated value. The expected *K*-function for a distribution following CSR is denoted (*E*[^∧^*K*(*t*)]). An estimation of ^∧^*K*(t) (or in general any stochastic quantity) is based on sample observations under given assumptions that might not always be fulfilled. One assumption in the present calculations is that neurons can occupy all of the sample area. The expectation (*E*[^∧^*K*(*t*)]) of a stochastic quantity is the mean value of the quantity under fulfilled assumptions over the entire population. “*t*” is the distance from an arbitrary cell and it determines the radius of the circle in which the cell intensity is calculated. To determine if the estimated *K*-functions calculated for a data set is non-random the fraction of the estimated *K*-functions (^∧^*K*) following CSR simulation that are further from the expected *K*-function *E*[^∧^*K* (*t*)] than the sample set's average ^∧^*K*-function is calculated. When the fraction is less than 0.05, we can discard randomness on a significance level of 0.05.

**Figure 1:**
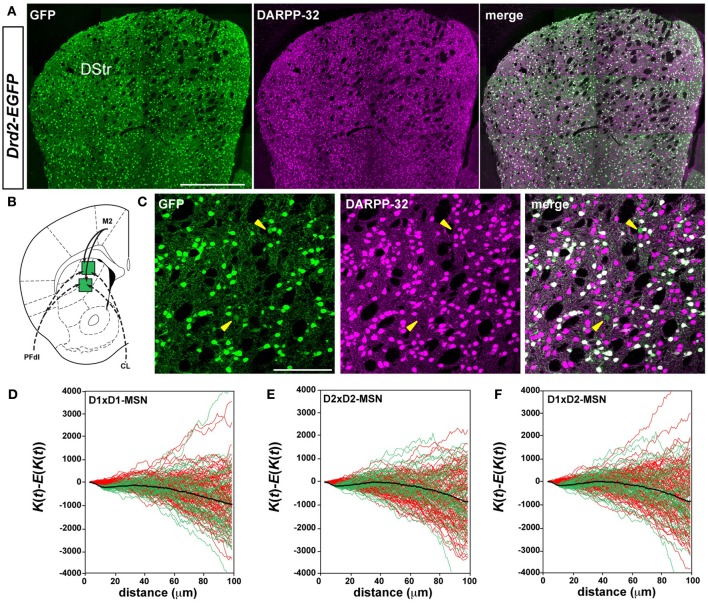
**D1R- and D2R-expressing MSNs are randomly distributed in the rostral part of the dorsal striatum**. **(A)** Low magnification showing the distribution of GFP (green) and DARPP-32 (magenta) a marker of MSNs in the dorsal striatum of *Drd2-EGFP* mice (*n* = 10). Scale bar, 500 μm. **(B)** Schematic illustration of 2 striatal sectors analyzed, known to receive inputs from M2 (motor cortex, solid lines), from two thalamic nuclei (dotted lines), PFdl (dorsolateral parafascicular nucleus) and CL (centrolateral nucleus). **(C)** Representative images showing DARPP-32 immunoreactivity (magenta) and GFP (green) in the dorsal striatum of *Drd2-EGFP* mice used for spatial distribution analysis. Yellow arrowheads identify putative ChAT interneurons (DARPP-32-negative, GFP-positive). Scale bar, 100 μm. **(D–F)** Analysis of Ripley's *K*-function for D1R-MSNs (GFP-negative) and D2R-MSNs (GFP-positive) distributions (green lines) with the average (black line) compared to simulated distributions following CSR (red lines). The overlap of green and red lines indicates that the experimental data do not differ from a distribution following CSR (this is also confirmed by statistical test, see Materials and Methods). Each green or red line is from the calculated *K*-function for one data sample area. The distribution of D1R-MSNs (D1×D1) **(D)**, D2R-MSNs (D2×D2) **(E)** and the spatial relation between D1R- and D2R-MSNs (D1×D2) (**F**) was random. DStr, dorsal striatum; GFP, green fluorescent protein.

The *K*-function analysis is displayed as the difference between the estimated *K*-function (^∧^*K*(t)) and the expected (E[^∧^*K*(t)]) *K*-function (^∧^*K*(t) − E[^∧^*K*(t)]) to make deviations from the CSR pattern more noticeable (Hansson et al., 2013). When the estimated *K*-function value is similar to the expected value for a distribution following CSR, denoted *E*[(^∧^*K*(*t*))] the difference (^∧^*K*(*t*) − *E*[^∧^*K*(*t*)]) is close to zero and we cannot discard that the sample distribution is following CSR (e.g., Figure 1D); when the difference is positive (the values of the estimated *K*-function are higher than the expected value from a distribution following CSR) it indicates aggregation; when the difference is negative it indicates inhibition, where events (cells) tend to repel other events (cells) (e.g., Figure 3D). Figures show the difference ^∧^*K*(*t*) − *E*[^∧^*K*(*t*)] as a function of distance “*t*” for all the distributions in the experimental data set (green lines) and all the simulated distributions following CSR (red lines) generated to compare with the experimental data. Simulated data had the same average density as the experimental data. In the figures each line (green or red) is derived from the calculated *K*-function from one data sample (neuron distribution sampled from 444 μm × 444 μm images).

## Results

### Striatonigral and striatopallidal MSNs are randomly distributed in the rostral part of the dorsal striatum

**Figure 2:**
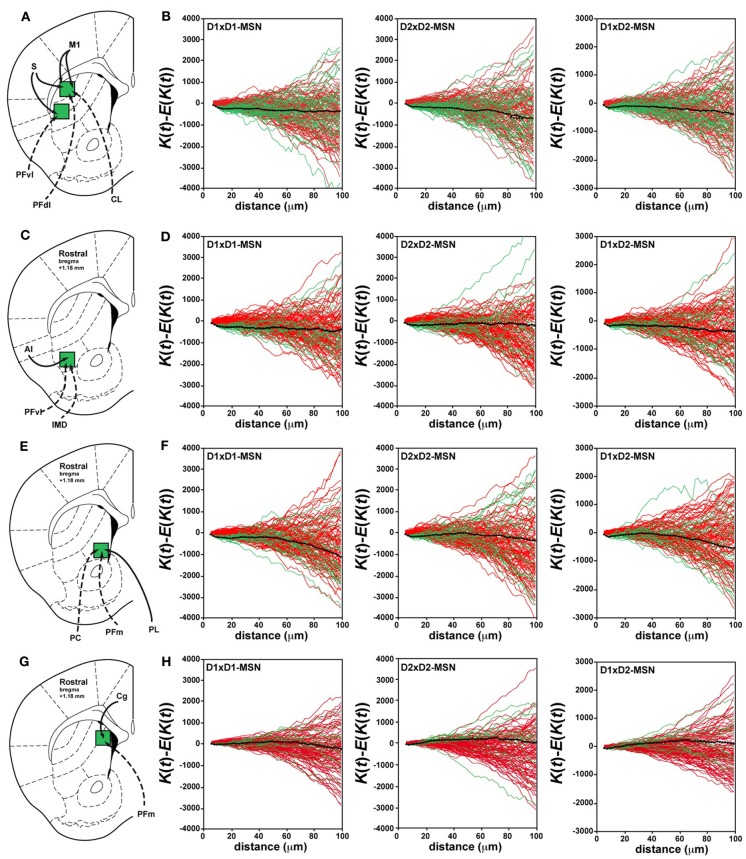
**Random distribution of D1R- and D2R-expressing MSNs in the rostral part of the dorsal striatum**. **(A,C,E,G)** Schematic illustration of the different striatal sectors analyzed at the rostral level with their corticostriatal (solid lines) and thalamostriatal (dotted lines) connections. **(A)** Striatal sectors receiving cortical inputs from M1 (motor cortex) and S (somatosensory cortex) and thalamic inputs from PFdl (dorsolateral parafascicular nucleus), PFvl (ventral lateral parafascicular nucleus) and CL (centrolateral nucleus). **(C)** Striatal sector connected preferentially by AI (agranular insular cortex), PFvl (ventral lateral parafascicular nucleus) and IMD (intermediodorsal nucleus). **(E)** Striatal sector connected by PL (prelimbic cortex), PFm (medial parafascicular nucleus) and PC (paracentral nucleus). **(G)** Striatal sector connected preferentially by Cg (cingulate cortex) and PFm (medial parafascicular nucleus). **(B,D,F,H)** Analysis of Ripley's *K*-function for D1R-MSNs (GFP-negative in *Drd2-EGFP* mice) and D2R-MSNs (GFP-positive in *Drd2-EGFP* mice) distributions (green lines) with the average (black line) compared to simulated distributions following CSR (red lines). The distribution of D1R-MSNs (D1×D1) (left column), D2R-MSNs (D2×D2) (middle column) and the spatial relation between D1R- and D2R-MSNs (D1×D2) (right column) was random. The overlap of green (test data) and red (simulated data) lines indicates that the experimental data do not differ from a distribution following CSR (this is also confirmed by statistical test, see Materials and Methods). Each green or red line is from the calculated *K*-function for one data sample area.

In the current scheme of the striatal organization, D1R- and D2R-expressing MSNs are thought to be intermingled throughout the striatum (Gerfen and Surmeier, 2011). To test this assumption, we investigated their spatial organization. D1R- and D2R-expressing MSNs were identified using BAC transgenic mice expressing the enhanced green fluorescent protein (EGFP) under the control of the promoter of Drd1a (*Drd1a-EGFP*) or Drd2 (*Drd2-EGFP*) combined with DARPP-32 immunoreactivity, which labels all MSNs (Figure 1A) (Matamales et al., 2009). Spatial point pattern statistics (Eglen and Wong, 2008; Jafari-Mamaghani et al., 2010) was used to analyze the distribution and the interrelation of cells in five striatal sectors of the rostral striatum (+1.18 mm relative to bregma, Figures 1B,C, 2). These sectors were chosen on the basis of tract-tracing studies (http://connectivity.brain-map.org/) stressing the existence of a topographic arrangement of the cortical and thalamic inputs, which target separate and specific functional striatal regions (Graybiel, 1984; Selemon and Goldman-Rakic, 1985; Haber et al., 2006; Gangarossa et al., 2013a).

We examined in these five striatal sectors the density of D1R- and D2R-expressing MSNs, a parameter that could contribute to differences in neuronal network topology. Taking into account the total surface of each striatal sector analyzed and averaging the results obtained from both *Drd1a*- and *Drd2-EGFP* mice (Table 1), we observed that the density of D1R-expressing MSNs was 10–20 % higher than the density of D2R-containing MSNs in the different areas analyzed, confirming previous observations (Bertran-Gonzalez et al., 2008; Matamales et al., 2009). The D1R-cell density was lower in striatal sectors known to receive massive projections from sensorimotor/motor cortex and from centrolateral (CL), ventral lateral parafascicular (PFvl), dorsolateral parafascicular (PFdl) and paracentral (PC) thalamic nuclei (http://connectivity.brain-map.org/), with a gradual increase moving toward the medial part of the striatum. Thus, the density of D1R-expressing cells was ~2-fold higher in medial striatal sectors innervated by cortical inputs from the cingulate cortex and thalamic projections from medial parafascicular (PFm) thalamic nucleus as compared to lateral striatal sectors (Table 1). The D2R-MSNs density was comparable between the areas with the exception of also being ~2-fold higher in medial striatal sectors (Table 1). Despite the difference in cell density, we found that in all the areas analyzed the spatial distribution of D1R- and D2R-expressing MSNs did not differ from simulated distributions following CSR (Figures 1D, 2). Figures 1D,E shows the analysis of Ripley's *K*-function for all the distributions in the experimental data set (green lines) and all the simulated distributions following CSR (red lines) generated to compare with experimental data. Each line is the results from one sample area. From visual inspection it can be inferred that if the *K*-functions for the distributions of the experimental data (green lines) and the simulated ones (red lines) overlap, the hypothesis that the experimental data is based on CSR cannot be discarded. Statistical analysis (see Materials and Methods; Jafari-Mamaghani et al., 2010; Hansson et al., 2013) of the *K*-functions from the experimental and the simulated data confirmed that the D1R- and D2R-MSNs populations are randomly distributed. Although D1R- and D2R-MSNs are randomly distributed they could be dependent on each other. We therefore analyzed interdependence between D1R- and D2R-MSNs according to the same principle using Ripley's *K*-function with the test distributions compared to each other (Eglen et al., 2005; Eglen and Wong, 2008). The superposition of the test data and the distributions following CSR suggest a lack of spatial correlation between the cell types (Figures 1F, 2). Altogether our analyses revealed the existence of different cell densities in the rostral part of the striatum. Moreover the spatial distribution analysis clearly showed that D1R- and D2R-containing MSNs are randomly distributed in the rostral part of the dorsal striatum.

**Table 1:**
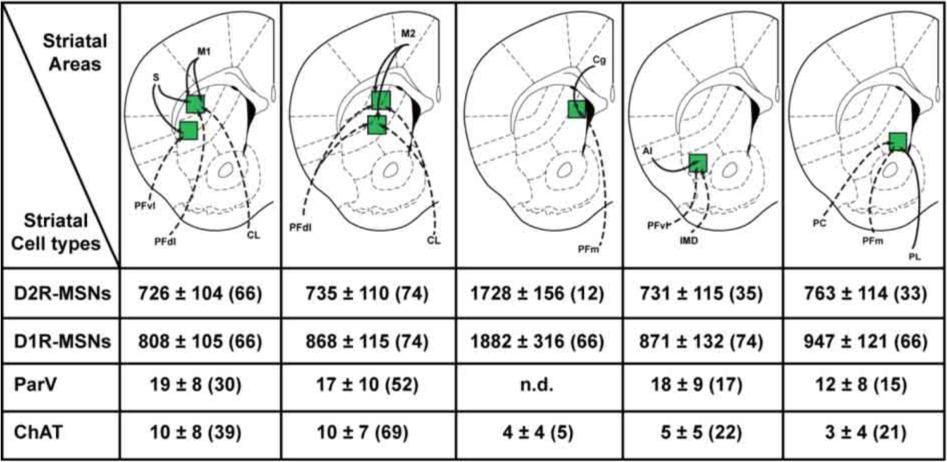
**Density of MSNs, parvalbumin GABAergic and cholinergic interneurons in the different striatal sectors analyzed at the rostral level of the dorsal striatum**.

*Cell density: cells/mm^2^. In parenthesis is the number of images (444 × 444 μm) that were used to make the calculations. Not determined (n. d.)*.

### Striatonigral and striatopallidal MSNs are anatomically segregated in the caudal part of the dorsal striatum

**Figure 3:**
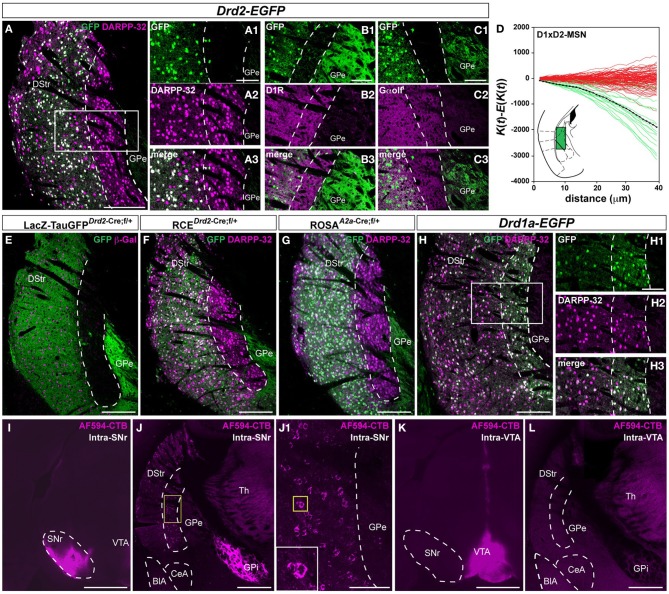
**Segregated distribution of D1R- and D2R/A2aR-expressing MSNs in the caudal part of the striatum**. **(A)** DARPP-32 (magenta) and GFP (green) immunofluorescence in the caudal part of the dorsal striatum of *Drd2-EGFP* mice (*n* = 10). Scale bar, 200 μm. High magnification of the area delineated by the white rectangle (GFP, **A_1_**; DARPP-32, **A_2_**; merge **A_3_**). Scale bar, 100 μm. Note the absence of D2R-expressing MSNs in a longitudinal stripe bordering the GPe. **(B,C)** Striatal sections from *Drd2-EGFP* mice (*n* = 5) stained with antibodies against D1R (GFP, **B_1_**; D1R, **B_2_**; merge, **B_3_**) and Gα olf (GFP, **C_1_**; DARPP-32, **C_2_**; merge **C_3_**). Scale bar, 100 μm. **(D)** Analysis of Ripley's *K*-function for D1R-MSNs (GFP-negative) and D2R-MSNs (GFP-positive) distributions (green lines) with the average (black line) compared to simulated distributions following CSR (red lines). For the experimental data (green lines) the difference between the estimated *K*-function and the expected *K*-function (^∧^*K*(*t*) − *E*[^∧^*K*(*t*)]) is negative indicating that the D1R- and D2R-MSNs occupy different parts of the sample area; in addition the distribution of the green lines does not overlap with the distribution of the simulated data (red lines) indicating a non-random distribution of D1R-MSNs and D2R-MSNs with respect to each other. Because of the non-random distribution, only the spatial relation between D1R- and D2R-positive MSNs (D1xD2) was evaluated. Inset, schematic illustration of the areas analyzed. **(E,F)** Pattern of Cre-mediated recombination visualized by immunofluorescence of GFP (green, **E** and **F**) and β-galactosidase (β-Gal; magenta, **E**) and DARPP-32 (magenta, **F**) in mice carrying both *Drd2-Cre* and *NLS-LacZ-Tau^mGFP^:LoxP* (*n* = 3) **(E)** or *RCE:LoxP* (*n* = 5) transgenes **(F)**. **(G)** Pattern of Cre-mediated recombination visualized by immunofluorescence staining using antibodies against GFP (green) and DARPP-32 (magenta) in carrying both *Adora2a-Cre* and *Rosa26:loxP* (*n* = 5) transgenes. Scale bar, 200 μm. **(H)** DARPP-32 (magenta) and GFP (green) immunofluorescence in the caudal part of the dorsal striatum of *Drd1a-EGFP* mice (*n* = 5). High magnification of the area delineated by the white rectangle (GFP, **H_1_**; DARPP-32, **H_2_**; merge **H_3_**). Scale bar, 100 μm. Note the complete co-localization of DARPP-32 and GFP in a longitudinal stripe bordering the GPe. (**I**) Injection site of AF594-CTB in the SNr. Scale bar, 1 mm. **(J)** After intra-SNr injection of AF594-CTB retrogradely labeled MSNs are located in the longitudinal stripe bordering the GPe. Scale bar, 500 μm. High magnification of the area delineated by the yellow square **(J_1_)**. Scale bar, 100 μm. **(K)** Injection site for AF594-CTB in the VTA. Scale bar, 1 mm. **(L)** MSNs located in the area lacking D2R/A2aR-expressing MSNs are not retrogradely labeled after AF594-CTB injection in the VTA. Scale bar, 500 μm. DStr, dorsal striatum; GPe, external globus pallidus; GFP, green fluorescent protein; Th, thalamus; GPi, internal globus pallidus; SNr, substantia nigra pars reticulata; VTA, ventral tegmental area; BlA, basolateral amygdala; CeA, central amygdala.

Because of the topographic arrangement of the cortical and thalamic inputs, we then analyzed the spatial distribution of D1R- and D2R-containing MSNs in the caudal part of the striatum (Gangarossa et al., 2013a). Unexpectedly, we identified in *Drd2-EGFP* mice a D2R-expressing MSNs-poor zone at the caudomedial margin of the striatum (−1.85 mm relative to bregma) (Figures 3, A2). Although a high density of DARPP-32-positive neurons was found in this striatal area, only few scattered MSNs co-localized with GFP expressed under the control of D2R promoter (Figures 3A, A2). Quantitative analysis revealed that ~4.9% of D2R-expressing neurons out of NeuN-positive cells were detected throughout the extension of this striatal area (292/5549 and 225/4928, *n* = 2 hemispheres). Moreover, no GFP staining was observed in the neuropil of *Drd2-EGFP* mice suggesting the absence of labeled dendrites and/or axons terminals of D2R-expressing neurons (Figure 3). In contrast, immunofluorescence using D1R antibody, which specifically labels the dendrites of D1R-expressing neurons, and Gα olf antibody, which recognizes the striatal-enriched G-protein expressed in MSNs, showed a dense staining confirming the presence of D1R-expressing MSNs in this striatal area (Figures 3B,C). Consequently, the analysis of the spatial distribution revealed that D1R- and D2R-expressing MSNs were physically segregated in this region of the striatum (Figure 3D). Indeed, analyses of Ripley's *K*-function showed that the relationship between the position of D1R- and D2R-MSNs is more dispersed than expected if the distributions were randomly distributed in relation to each other (Figure 3D).

This anatomical segregation was confirmed in *Drd2-Cre* BAC transgenic mice crossed with the *Tau^mGFP^* reporter line, which leads to the expression of nuclear β-galactosidase and membrane-targeted GFP (mGFP) (Hippenmeyer et al., 2005). In the double transgenic mice, β-galactosidase-positive cells and their GFP-positive terminals were confined to the lateral-caudal part of the dorsal striatum as observed in *Drd2-EGFP* mice (Figure 3E). Interestingly, the very low GFP staining indicates a scarcity of D2R-containing dendrites or terminals in contrast to other striatal regions. Similar results were obtained when we crossed *Drd2-Cre* mice with the *RCE:LoxP* reporter line (Miyoshi et al., 2010) (Figures 3F, A2). The perfect overlap of the GFP staining observed in the three lines demonstrates that this expression most likely reflects the activity pattern of the endogenous promoter. Importantly, a similar spatial distribution of MSNs was also observed when *A2a-Cre* BAC transgenic mice crossed with the *Rosa26:LoxP* reporter line were used to identify striatopallidal neurons (Durieux et al., 2009) (Figure 3G). Only few scattered A2aR-expressing neurons (~3.8%) were observed among NeuN-positive cells throughout the extension of this caudal region of the striatum (221/6028 and 240/6245, *n* = 2 hemispheres), thus confirming the results obtained using the *Drd2-EGFP* mouse line. As for the *Drd2-EGFP* or *Drd2-Cre* mice, no GFP staining was found in the neuropil of *A2a-Cre* mice suggesting the absence of labeled dendrites of A2aR-expressing neurons (Figure 3G). In contrast, in *Drd1a-EGFP* mice the vast majority of DARPP-32-positive cells were GFP-positive suggesting that this striatal region is composed mostly of D1R-expressing MSNs (Figure 3H). Using retrograde labeling, we then examined whether D1R-expressing MSNs located in this striatal area were striatonigral MSNs. Alexa Fluor (AF594) conjugate of cholera toxin subunit B (CTB), a fluorescent retrograde tracer, was stereotactically injected into the SNr (Figure 3I). AF594-CTB-immunoreactive neurons were detected at the caudomedial margin of the striatum (Figure 3J). Because we will never be able to ascertain that the retrograde tracer is taken exclusively by striatopallidal inputs and not by the projections of D1-MSNs that terminate selectively or en passant into the GPe, the injection of AF594-CTB directly into the GPe has not been performed. Future studies using single axon reconstruction should help to clarify whether D1R-expressing MSNs located in this area also project to the GPe. Finally, the lack of labeling when the AF594-CTB was injected in the VTA strongly suggests that this striatal region is composed preferentially of striatonigral MSNs (Figures 3K,L).

**Figure 4:**
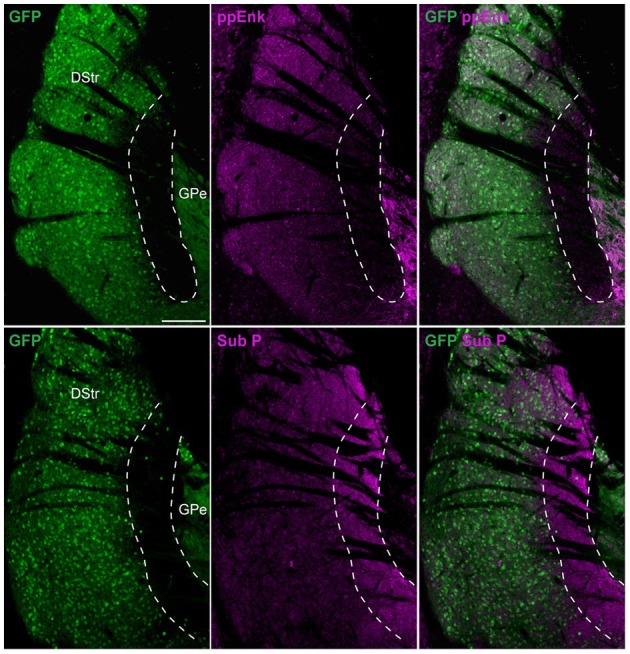
**Preproenkephalin and substance P immunoreactivities in the D2R/A2aR-expressing MSNs-poor zone of the caudal striatum**. Striatal sections at the caudal level from *Drd2-EGFP* mice (*n* = 5) labeled with antibodies for preproenkephalin (ppEnk, magenta) or substance P (Sub P, magenta) and GFP (green). Note the very low level and the enrichment of preproenkephalin- and substance P-labeling, respectively in the area lacking D2R/A2aR-expressing MSNs. DStr, dorsal striatum; GPe, external globus pallidus, GFP, green fluorescent protein.

These observations suggest the intriguing possibility that striatopallidal MSNs are very rare in this striatal area. Since D2R and A2aR are just two among many molecular markers that are selectively expressed by striatopallidal MSNs, we also tested the immunoreactivity of preproenkephalin (ppEnk), the enkephalin precursor, which is a well-known marker of striatopallidal MSNs. The absence of enkephalin-positive fibers in this area strongly supports the paucity of striatopallidal MSNs and rules out the possibility of a mismatch between the expression of D2R/A2aR and enkephalin (Figure 4). Moreover, the enrichment of substance P (Sub P)-positive fibers, a well-known marker of striatonigral MSNs further suggest that this area comprises mostly striatonigral D1R/Sub P MSNs (Figure 4).

### Anatomical and neurochemical characterization of the D2R/A2aR-expressing MSNs-poor zone of the caudal striatum

**Figure 5:**
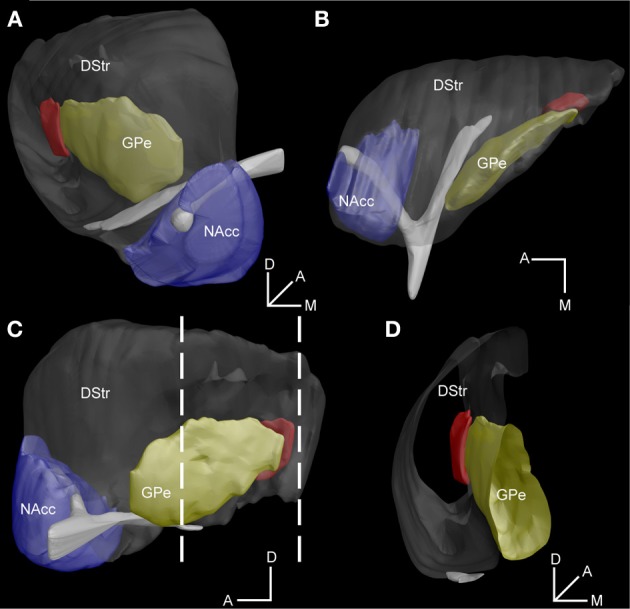
**3D reconstruction model of the D2R-expressing MSNs-poor zone of the caudal striatum**. **(A–C)** Illustration of the striatum within the global model as examined from frontal **(A)**, parahorizontal **(B)** and sagittal **(C)** views of the model. **(D)** Virtual coronal slice of the striatum at the level indicated by the stippled lines in **C**. The DStr, dorsal striatum in gray, NAcc, nucleus accumbens in blue, GPe, external globus pallidus in yellow and the area lacking striatopallidal D2R/A2aR MSNs in red. Orientation axes: D, dorsal, A, anterior, M, medial.

**Figure A1:**
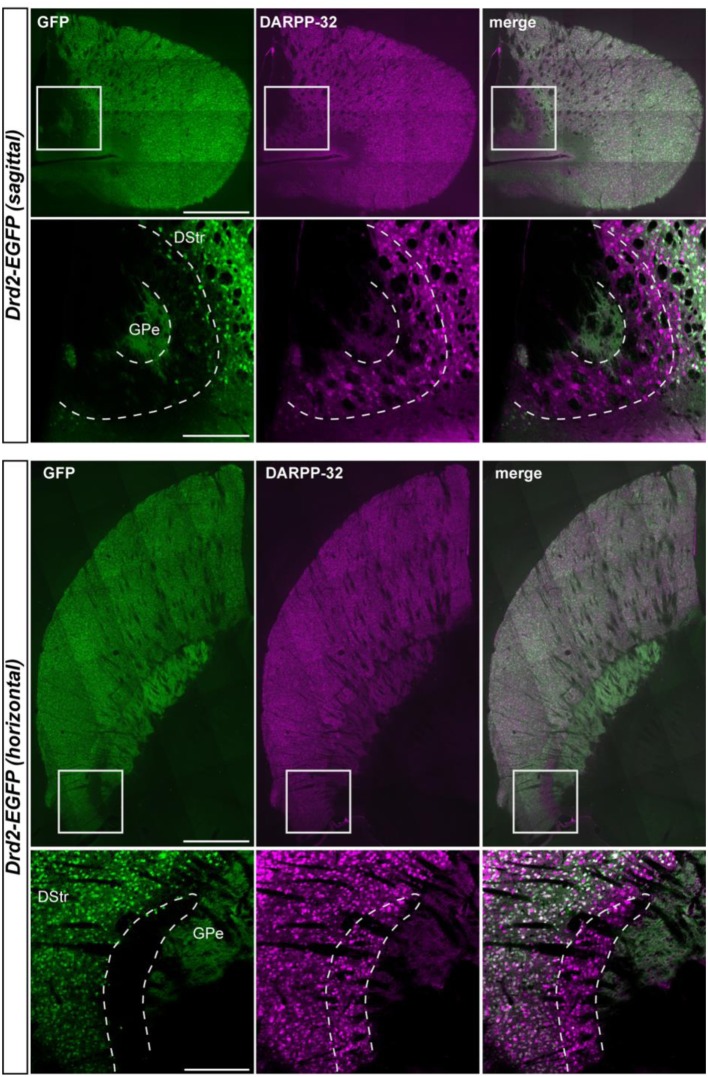
**Whole-striatum anatomical characterization of the D2R/A2aR-expressing MSNs-poor zone of the caudal striatum**. Sagittal **(upper panel)** and parahorizontal **(lower panel)** view of the striatum of *Drd2-EGFP* mice immunostained for DARPP-32 (magenta). Scale bars: 1 mm. Insets, higher magnification of the striatal area located at the caudomedial margin of the striatum empty of D2R-expressing MSNs and stained only with DARPP-32. Scale bars, 200 μm. DStr, dorsal striatum; GPe, external globus pallidus, GFP, green fluorescent protein.

**Figure A2:**
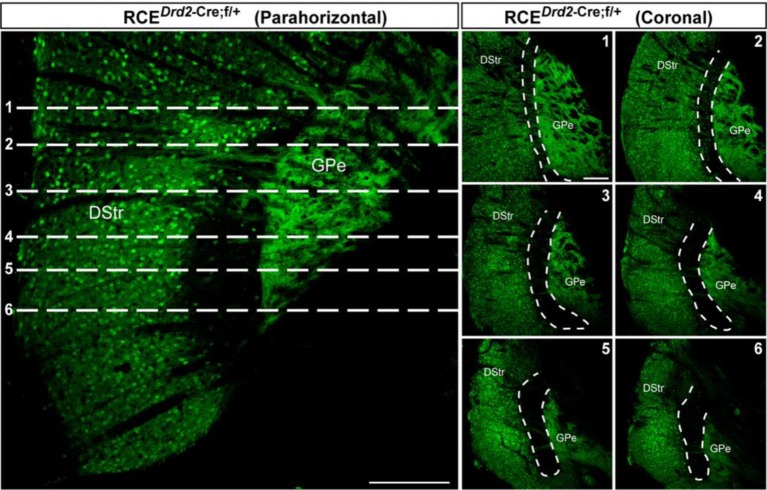
**Rostro-caudal extension of the the D2R/A2aR-expressing MSNs-poor zone of the caudal striatum**. **(left panel)** GFP immunofluorescence in a parahorizontal section of *Drd2-Cre* crossed with the reporter line *RCE:LoxP*. Scale bar, 200 μm. **(right panels)** Frontal view of the striatum corresponding to the level indicated by the stippled lines (1–6) presented on the parahorizontal view. Scale bar, 200 μm. Note that the size of the D2R/A2aR-expressing MSNs-poor zone progressively increases along the rostro-caudal extension. DStr, dorsal striatum; GPe, external globus pallidus, GFP, green fluorescent protein.

To more precisely locate this region within the entire striatum, we built a 3D reconstruction model based on series of coronal GFP stained sections from *Drd2-EGFP* mice aligned to blockface pictures (Mailly et al., 2009). As illustrated in Figure 5, this specific striatal region (in red) appears as a longitudinal stripe (volume = 0.041 mm^∧^3, rostrocaudal extension = 0.51 mm) located at the caudomedial margin of the striatum surrounding the caudolateral edge of the external globus pallidus (GPe) (Figures 5, A1).

**Figure 6:**
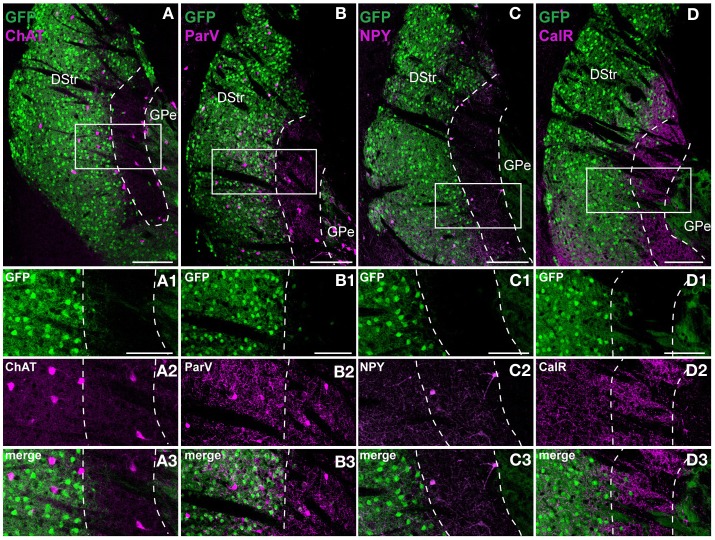
**Striatal interneurons distribution in the D2R/A2aR-expressing MSNs-poor zone of the caudal striatum**. Striatal sections at the caudal level from *Drd2-EGFP* mice (*n* = 5 for each staining) labeled with antibodies for choline acetyltransferase (ChAT, **A**), parvalbumin (ParV, **B**), neuropeptide Y (NPY, **C**) and calretinin (CalR, **D**). Scale bars, 200 μm. Insets, higher magnification (GFP, green, **A_1_**; ChAT, magenta, **A_2_**; merge, **A_3_**), (GFP, green, **B_1_**; ParV, magenta, **B_2_**; merge, **B_3_**), (GFP, green, **C_1_**; NPY, magenta, **C_2_**; merge **C_3_**) and (GFP, green, **D_1_**; CalR, magenta, **D_2_**; merge **D_3_**). Scale bars, 100 μm. DStr, dorsal striatum; GPe, external globus pallidus, GFP, green fluorescent protein.

We then performed an in-depth characterization of the neurochemical profile of this striatal area. In *Drd2-EGFP* mice, GFP is present in DARPP-32-positive cells as well as in ChAT-positive interneurons (Bertran-Gonzalez et al., 2008; Matamales et al., 2009). Thus, we found that at the rostral level of the striatum 267 out of 285 ChAT-positive cells co-expressed GFP (~94% of co-localization). Interestingly, when the same analysis was performed in the striatal area lacking D2R/A2aR MSNs, only 13 out of 71 ChAT interneurons showed a weak expression of GFP (~17% of co-localization) (Figure 6A). We estimated the percentage of cholinergic and GABAergic interneurons throughout the extension of this area. Our analysis revealed that this region of the caudal striatum contained ~3.1% of ChAT-positive interneurons (201/6137 and 153/5239, *n* = 2 hemispheres), ~2.7% of NPY-positive interneurons (154/5549 and 132/4928, *n* = 2 hemispheres) and ~1.1% parvalbumin-positive interneurons (69/6137 and 51/5239, *n* = 2 hemispheres) (Figures 6B,C) (Kawaguchi, 1993, 1997; Tepper et al., 2010). Finally, although really few calretinin-positive interneurons were detected ~0.4% (23/6028 and 20/6245, *n* = 2 hemispheres), dense calretinin-positive fibers were observed (Figure 6D).

**Figure 7:**
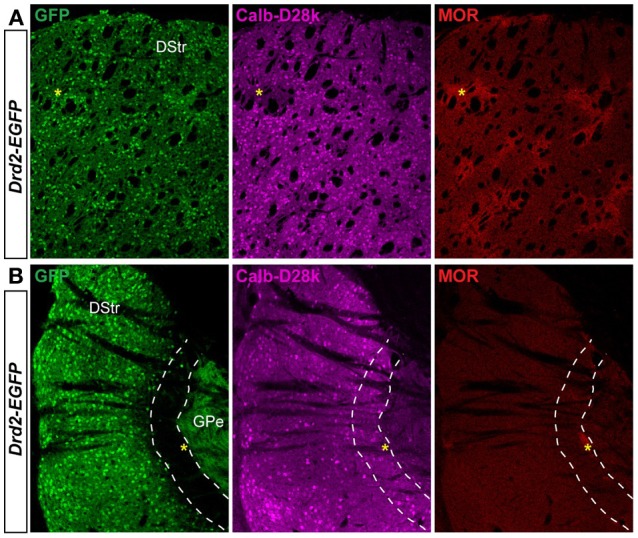
**Patch/matrix organization in the D2R/A2aR-expressing MSNs-poor zone of the caudal striatum**. Striatal sections at the rostral **(A)** and the caudal **(B)** level from *Drd2-EGFP* mice (*n* = 3) labeled with antibodies for calbindin-D28k (Calb-D28k, middle panel) and μ opioid receptor (MOR, right panel) to identify matrix and striosome compartments, respectively. Note the presence of dense MOR staining identifying striosomes in the rostral part of the striatum (yellow asteriks). Scale bars, 200 μm. DStr, dorsal striatum; GPe, external globus pallidus, GFP, green fluorescent protein.

We next assessed whether this striatal area displayed particular features regarding the striosome/matrix compartmentalization. Calbindin-D28k and μ opiate receptor (MOR) immunoreactivities were used to identify the matrix and the striosomal compartments, respectively (Figure 7A) (Herkenham and Pert, 1981; Liu and Graybiel, 1992; Davis and Puhl, 2011). As shown in Figure 7B, this specific area of the caudal striatum expresses calbindin-D28k and is poor in MOR suggesting that this region could be similar to a matrix area.

**Figure 8:**
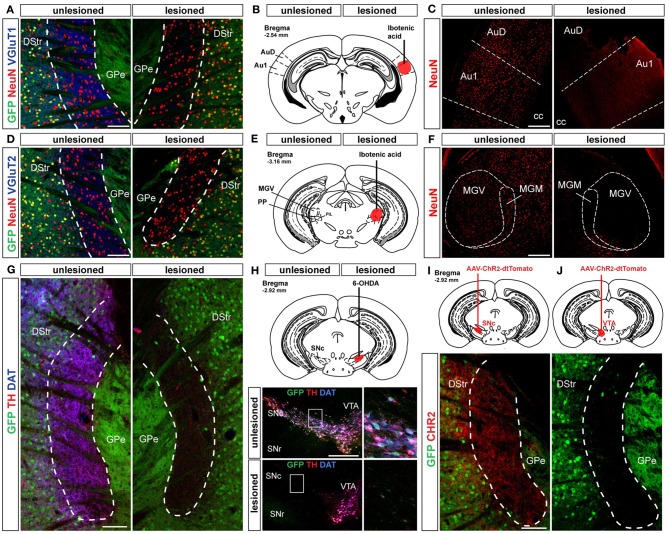
**Cortical, thalamic and dopaminergic inputs to the D2R/A2aR-expressing MSNs-poor zone of the caudal striatum**. **(A)** Triple immunostaining GFP (green), NeuN (red) and VGluT1 (blue) allowing the identification of corticostriatal inputs. Scale bar: 100 μm. **(B)** Localization of the lesion induced by ibotenic acid microinjection in the primary auditory cortex (bregma, −2.54 mm). **(C)** NeuN (red) immunoreactivity in auditory cortex of *Drd2-EGFP*-lesioned mice (*n* = 3). **(D)** Triple immunostaining GFP (green), NeuN (red) and VGluT2 (blue) allowing the identification of thalamostriatal inputs. Scale bar: 100 μm. **(E)** Localization of the lesion induced by ibotenic acid microinjection in the medial geniculate thalamic nucleus (bregma, −3.08 mm). **(F)** NeuN (red) immunoreactivity in medial geniculate thalamic of *Drd2-EGFP*-lesioned mice (*n* = 3). Note the loss of NeuN positive neurons on the lesioned sides **(C,F)**. **(G)** Triple immunostaining GFP (green), TH (red) and DAT (blue) at the level of the striatal area lacking D2R/A2aR MSNs. Scale bar: 100 μm. **(H)** Localization of the 6-OHDA lesion in the SNc site (bregma, −2.92 mm). Triple immunostaining GFP (green), TH (red) and DAT (blue) allowed the identification of DA neurons on the unlesioned side of *Drd2-EGFP* mice (*n* = 3). Scale bar: 300 μm. **(I,J)** Injection sites for the AAV-ChR2-dtTomato in the SNc (*n* = 2) **(I)** and the VTA (*n* = 2) **(J)**. AAV-ChR2-dtTomato-labeled terminals in the striatal area lacking D2R/A2aR MSNs. arise from the SNc but not from the VTA. Scale bar: 100 μm. DStr, dorsal striatum; GPe, external globus pallidus; GFP, green fluorescent protein; Au1, primary auditory cortex; AuD, auditory cortex dorsal part; MGV, medial geniculate ventral; MGM, medial geniculate median; SNr, substantia nigra pars reticulata; SNc, substantia nigra pars compacta; VTA, ventral tegmental area.

Striatal MSNs integrate the glutamatergic afferents arising from the cortex and the thalamus that differentially express the vesicular transporter type 1 (VGluT1) or type 2 (VGluT2), respectively (Fremeau et al., 2004). We found that both markers were highly expressed in the D2R/A2aR-expressing MSNs-poor zone (Figures 8A,D) indicating that D1R-expressing MSNs in this area receive synaptic inputs from both the cerebral cortex and the thalamus. Earlier tract-tracing studies performed in rats identified the primary auditory cortex and the medial geniculate nucleus as major sources of cortical and thalamic inputs to the caudal part of the dorsal striatum (Ledoux et al., 1991). Lesions of the primary auditory cortex (Figures 8A–C) and the medial geniculate nucleus (Figures 8D–F) using ibotenic acid were accompanied by a loss of VGluT1 (Figure 8A) and VGluT2 (Figure 8D) immunoreactivities in the D2R/A2aR-expressing MSNs-poor zone of the caudal striatum confirming the conservation of this connectivity in the mouse.

Whereas the glutamatergic inputs activate striatal circuits, the dopaminergic inputs arising from the SNc have a crucial modulatory role. Thus, a dense lattice of TH (tyrosine hydroxylase) and DAT (dopamine transporter) immunopositive terminals was observed in the D2R/A2aR-expressing MSNs-poor zone of the caudal striatum (Figure 8G). Both stainings disappeared following ipsilateral intra-SNc 6-OHDA lesion (Figure 8H). Moreover, microinjection of an AAV encoding ChR2-dtTomato used as an anterograde tracer in the SNc or the VTA confirmed that this area is densely innervated by dopaminergic inputs from the SNc (Figures 8I,J).

## Discussion

The current view of the anatomical organization of the striatum assumes that “striatonigral” MSNs (D1R/substance P) and “striatopallidal” MSNs (D2R/A2aR/enkephalin) are mostly intermixed throughout the striatum (Gerfen and Surmeier, 2011). The present study confirmed this hypothesis for a large part of this structure. However, we identified a specific region located in the caudal striatum, which in contrast was composed of mostly D1R-expressing MSNs.

### Anatomical segregation of D1R- and D2R/A2aR-expressing MSNs in a specific and restricted region of the caudal striatum

Using various transgenic mice allowing BAC-driven expression of EGFP (*Drd1a-EGFP*, *Drd2-EGFP*) or Cre recombinase (*Drd2-cre*, *Adora2a-Cre*), we identified a D2R/A2aR-expressing MSNs-poor striatal subdivision located at the caudomedial margin of the striatum (appromimatively from −1.46 to −2.06 mm relative to bregma). It is important to mention that the same anatomical profile has been observed in *Drd2*- and *Drd1a-EGFP* mice independently of the strains (*Drd2-EGFP* vs. *Drd2-Cre*) and the background (Swiss-Webster vs. C57BL/6N). In addition, the *Adora2a-Cre* mice (C57BL/6N) displayed the same pattern, which make us feel confident about the fact that the different mouse strains used do not introduce any strain-dependent differences. This small area (see 3D model, Figure 5) comprises almost exclusively D1R-expressing MSNs whereas only few D2R/A2aR-expressing MSNs were found. Moreover, the virtual absence of GFP-positive neuropil in *Drd2-EGFP*, *Drd2-Cre*, and *Adora-Cre* BAC reporter mice suggests this striatal region does not have D2R/A2aR modulation. Our findings provide an explanation for the lack of the glycoprotein ecto-5′-nucleotidase (NT5e) staining reported earlier in the caudal part of the mouse striatum (Schoen and Graybiel, 1993). Indeed, NT5e, which hydrolyzes adenosine-5′ monophosphate to adenosine (Lee et al., 1986), has been recently found to be selectively expressed in D2R- and A2aR-expressing MSNs [see Table S2 in Heiman et al. (2008); Ena et al. (2013)].

An important issue is related to the projections of neurons in the caudomedial margin of the striatum. We show here that, as in other striatal regions (Gerfen et al., 1990; Gertler et al., 2008; Matamales et al., 2009), the MSNs in this region project massively to the SNr. However, we do not know whether these MSNs are “pure” striatonigral neurons or whether they also innervate the GPe to some extent. Interestingly, anatomical evidences suggest that a pure striatonigral pathway may not exist as such. Indeed, single-cell juxtacellular labeling studies in rats revealed that in contrast to the striatopallidal (D2R/A2aR) MSNs (type I) that project exclusively to GPe, most if not all the striatonigral (D1R) MSNs (type II) send projections to the GPe on their way to the SNr (Kawaguchi et al., 1990; Wu et al., 2000). The type II MSNs comprises the type IIa (also defined as type III) striatonigral neurons that project to GPe, GPi and terminate rather focally in the SNr, and the type IIb (or type II) MSNs that send axons to GPe, but not to GPi, and arborize more profusely and more widely in the SNr (Kawaguchi et al., 1990; Wu et al., 2000; Levesque et al., 2003). These data in rat are also in agreement with findings in BAC-transgenic mice expressing GFP (Matamales et al., 2009). Although, future studies will be necessary to determine whether this striatal area is composed of type IIa and/or IIb MSNs, our observations and previous findings in rats are compatible with the hypothesis that most MSNs located in this striatal area should in all likelihood innervate the GPe.

### Neurochemical features of D2R/A2aR-expressing MSNs-poor zone

There is already some evidence that “striatonigral” (D1R/substance P) and “striatopallidal” MSNs (D2R/A2aR/enkephalin) are not equally distributed in all dorsal striatal territories. Indeed, several authors reported a paucity of enkephalin-expressing MSNs in the striosomal compartments, as compared to matrix, in the rostral part of dorsal striatum (Graybiel and Chesselet, 1984; Koshimizu et al., 2008). The presence of Calb-D28k, TH, and VGluT2 immunoreactivities and the paucity of MOR and ppEnk stainings strongly suggest that this caudomedial striatal region corresponds to matrix-like compartment. However, this striatal area is also rich in Gα olf and calretinin, which are both denser in striosomes (Sako et al., 2010; Davis and Puhl, 2011). Taken together, our findings suggest that the neurochemical profile of this region is unique and additional studies will be necessary to further characterize whether this striatal area corresponds more to striosome or matrix compartments.

Although interneurons comprise only ~5% of all striatal neurons in rodents, they play an important role in regulating striatal MSNs (Kawaguchi, 1997; Tepper et al., 2010; Gittis and Kreitzer, 2012). The detection of parvalbumin- and NPY-positive cells, that allows the identification of FSIs and PLTSs interneurons (Kawaguchi, 1993), reveals the presence of inhibitory microcircuits in the D2R/A2aR-expressing MSNs-poor zone of the caudal striatum. In addition to GABAergic interneurons, we found that this striatal area also comprises large aspiny ChAT-positive interneurons. In the rostral part of the dorsal striatum, ChAT interneurons express D2R (94%) and can be easily identified in *Drd2-EGFP* mice (Bertran-Gonzalez et al., 2008; Matamales et al., 2009). In contrast, our data reveal that in this area of the caudal striatum, only ~17% of ChAT interneurons express GFP under the control of the D2R promoter. Altogether, these observations raise the intriguing possibility that striatal ChAT interneurons do not constitute a homogeneous cell population but rather comprise at least two sub-classes, characterized by distinct neurochemical features and differentially distributed along the rostro-caudal axis of the striatum.

### Excitatory inputs to the D2R/A2aR-expressing MSNs-poor zone of the caudal striatum

The information that MSNs process within the circuits of basal ganglia is largely determined by distributed patterns of cortical activity (Gerfen and Surmeier, 2011). Topographically organized, the corticostriatal inputs have been categorized into at least two different subgroups according to their laminar distribution in the cortex and their projections (Reiner et al., 2003; Shepherd, 2013). The intratelencephalic (IT)-type neurons give rise to bilateral corticocortical and corticostriatal projections (for review see Shepherd, 2013). Those neurons have been proposed to convey sensory and motor planning information to the striatum (Reiner et al., 2010) and to target preferentially striatonigral (D1R) MSNs (Lei et al., 2004; but see Ballion et al., 2008). On the other hand, the pyramidal tract (PT)-type neurons provide major projections directly to motor neurons in the brainstem and spinal cord, as well as collaterals to the striatum. These neurons would convey an efferent copy of motor commands and contact preferentially striatopallidal (D2R/A2aR) MSNs (Lei et al., 2004). There is also anatomical evidence indicating that IT and PT could have differential inputs to the striosome vs. matrix compartments (Crittenden and Graybiel, 2011). If the specificity of the corticostriatal connectivity described above is correct and conserved throughout the dorsal striatum, our data imply that this striatal area, which receives glutamatergic inputs from the auditory cortex, should be preferentially innervated by IT-type neurons. Although a more comprehensive map of cortical inputs is needed, future studies using mice allowing the identification of PT-type and IT-type neurons in the auditory cortex (Groh et al., 2010) will be useful to reappraise the PT and IT inputs in this striatal area.

### Does this striatal region correspond to the “marginal division”?

Despite the lack of easily identified striatal cytoarchitecture, previous studies have pointed out the existence of a subdivision in the most caudal part of the dorsal striatum termed the “marginal division” (MrD). This region, well characterized in rats, forms a longitudinal stripe bordering the GPe along the rostro-caudal axis (Shu et al., 1988). The MrD exhibits some singular features including densely packed fusiform neurons (Shu et al., 1988) as well as high densities of immunoreactive terminals of a variety of neuropeptides including L-Enkephalin, substance P, neurotensin, somatostatin, cholecystokinin and dynorphin B (Shu et al., 1988, 1990, 1999). Interestingly, the best hallmark distinguishing the MrD from the other parts of the striatum is so far the lack of the striatopallidal-enriched protein, NT5e (Schoen and Graybiel, 1993; Ena et al., 2013). After a careful reviewing of the molecular markers defining the MrD, it is difficult to ascertain that the D2R/A2aR-expressing MSNs-poor zone of the caudal striatum and the MrD are one and the same subdivision. Indeed, while the lack of NT5e staining and the enrichment in substance P in the caudal striatum strongly support this idea, the paucity of MOR and ppEnk immunoreactivities, argues against it. Several explanations could account for such differences. First, although there are common features, the rat and mouse MrD could differ slightly in their molecular profiles. Alternatively, the D2R/A2aR-expressing MSNs-poor zone of the caudal striatum would constitute a subdivision of the MrD. If this is the case, this implies that the neurochemical profile of the MrD and the molecular phenotype of its MSNs differ along the rostro-caudal axis. Whatever the reason, there is clearly a need to further characterize anatomically and functionally the MrD.

In conclusion, we demonstrate the existence of a specific region in the caudal striatum, adjacent to the GPe, which weakly expresses markers for indirect pathway neurons. This provides an example of a region of the dorsal striatum with low D2R expression, as we have recently reported in the nucleus accumbens shell (Gangarossa et al., 2013b). Altogether, our study highlights another level of heterogeneity within the striatum whose function remains to be established.

### Conflict of interest statement

The authors declare that the research was conducted in the absence of any commercial or financial relationships that could be construed as a potential conflict of interest.

## References

[B1] Ballion B. Mallet N. Bezard E. Lanciego J. L. Gonon F. (2008). Intratelencephalic corticostriatal neurons equally excite striatonigral and striatopallidal neurons and their discharge activity is selectively reduced in experimental parkinsonism. Eur. J. Neurosci. 27, 2313–2321 10.1111/j.1460-9568.2008.06192.x 18445222

[B2] Bertran-Gonzalez J. Bosch C. Maroteaux M. Matamales M. Hervé D. Valjent E. (2008). Opposing patterns of signaling activation in dopamine D1 and D2 receptor-expressing striatal neurons in response to cocaine and haloperidol. J. Neurosci. 28, 5671–5685 10.1523/JNEUROSCI.1039-08.2008 18509028 PMC6670792

[B3] Bertran-Gonzalez J. Hervé D. Girault J. A. Valjent E. (2010). What is the degree of segregation between striatonigral and striatopallidal projections. Front. Neuroanat. 4:136 10.3389/fnana.2010.00136 20953289 PMC2955397

[B4] Bolam J. P. Hanley J. J. Booth P. A. Bevan M. D. (2000). Synaptic organisation of the basal ganglia. J. Anat. 196(Pt 4), 527–542 10923985 10.1046/j.1469-7580.2000.19640527.x PMC1468095

[B5] Crittenden J. R. Graybiel A. M. (2011). Basal Ganglia disorders associated with imbalances in the striatal striosome and matrix compartments. Front. Neuroanat. 5:59 10.3389/fnana.2011.00059 21941467 PMC3171104

[B6] Davis M. I. Puhl H. L. 3rd. (2011). Nr4a1-eGFP is a marker of striosome-matrix architecture, development and activity in the extended striatum. PLoS ONE 6:e16619 10.1371/journal.pone.0016619 21305052 PMC3030604

[B7] Diggle P. J. (2003). Statistical Analysis of Spatial Point Patterns. London: Hodder Education Publishers

[B8] Durieux P. F. Bearzatto B. Guiducci S. Buch T. Waisman A. Zoli M. (2009). D2R striatopallidal neurons inhibit both locomotor and drug reward processes. Nat. Neurosci. 12, 393–395 10.1038/nn.2286 19270687

[B9] Durieux P. F. Schiffmann S. N. de Kerchove d'Exaerde A. (2011). Differential regulation of motor control and response to dopaminergic drugs by D1R and D2R neurons in distinct dorsal striatum subregions. EMBO J. 31, 640–653 22068054 10.1038/emboj.2011.400 PMC3273396

[B10] Eglen S. J. Diggle P. J. Troy J. B. (2005). Homotypic constraints dominate positioning of on- and off-center beta retinal ganglion cells. Vis. Neurosci. 22, 859–871 10.1017/S0952523805226147 16469193 PMC1513157

[B11] Eglen S. J. Wong J. C. (2008). Spatial constraints underlying the retinal mosaics of two types of horizontal cells in cat and macaque. Vis. Neurosci. 25, 209–214 10.1017/S0952523808080176 18334045

[B12] Ena S. L. De Backer J. F. Schiffmann S. N. de Kerchove d'Exaerde A. (2013). FACS array profiling identifies Ecto-5′ nucleotidase as a striatopallidal neuron-specific gene involved in striatal-dependent learning. J. Neurosci. 33, 8794–8809 23678122 10.1523/JNEUROSCI.2989-12.2013 PMC6618839

[B13] Franklin K. Paxinos G. (2007). The Mouse Brain in Stereotaxic Coordinates, 3rd Edn. Amsterdam: Elsevier

[B14] Frazer A. (2000). Norepinephrine involvement in antidepressant action. J. Clin. Psychiatry 61, 25–30 10910014

[B15] Fremeau R. T. Jr. Voglmaier S. Seal R. P. Edwards R. H. (2004). VGlUTs define subsets of excitatory neurons and suggest novel roles for glutamate. Trends Neurosci. 27, 98–103 10.1016/j.tins.2003.11.005 15102489

[B16] Fulceri F. Biagioni F. Lenzi P. Falleni A. Gesi M. Ruggieri S. (2006). Nigrostriatal damage with 6-OHDA: validation of routinely applied procedures. Ann. N.Y. Acad. Sci. 1074, 344–348 10.1196/annals.1369.032 17105931

[B17] Gangarossa G. Perroy J. Valjent E. (2013a). Combinatorial topography and cell-type specific regulation of the ERK pathway by dopaminergic agonists in the mouse striatum. Brain Struct. Funct. 218, 405–419 10.1007/s00429-012-0405-6 22453353

[B18] Gangarossa G. Espallergues J. de Kerchove d'Exaerde A. El Mestikawy S. Gerfen C. R. Hervé D. (2013b). Distribution and compartmental organization of GABAergic medium-sized spiny neurons in the mouse nucleus accumbens. Front. Neural Circuits 7:22 10.3389/fncir.2013.00022 23423476 PMC3575607

[B19] Gerfen C. R. (1989). The neostriatal mosaic: striatal patch-matrix organization is related to cortical lamination. Science 246, 385–388 10.1126/science.2799392 2799392

[B20] Gerfen C. R. Engber T. M. Mahan L. C. Susel Z. Chase T. N. Monsma F. J. (1990). D1 and D2 dopamine receptor-regulated gene expression of striatonigral and striatopallidal neurons. Science 250, 1429–1432 10.1126/science.2147780 2147780

[B21] Gerfen C. R. Surmeier D. J. (2011). Modulation of striatal projection systems by dopamine. Annu. Rev. Neurosci. 34, 441–466 10.1146/annurev-neuro-061010-113641 21469956 PMC3487690

[B22] Gerfen C. R. Young W. S. 3rd. (1988). Distribution of striatonigral and striatopallidal peptidergic neurons in both patch and matrix compartments: an *in situ* hybridization histochemistry and fluorescent retrograde tracing study. Brain Res. 460, 161–167 10.1016/0006-8993(88)91217-6 2464402

[B23] Gertler T. S. Chan C. S. Surmeier D. J. (2008). Dichotomous anatomical properties of adult striatal medium spiny neurons. J. Neurosci. 28, 10814–10824 10.1523/JNEUROSCI.2660-08.2008 18945889 PMC3235748

[B24] Gittis A. H. Kreitzer A. C. (2012). Striatal microcircuitry and movement disorders. Trends Neurosci. 9, 557–564 10.1016/j.tins.2012.06.008 22858522 PMC3432144

[B25] Goldman-Rakic P. S. (1982). Cytoarchitectonic heterogeneity of the primate neostriatum: subdivision into Island and Matrix cellular compartments. J. Comp. Neurol. 205, 398–413 10.1002/cne.902050408 7096628

[B26] Gong S. Doughty M. L. Harbaugh C. R. Cummins A. Hatten M. E. Heintz N. (2007). Targeting CRE recombinase to specific neurons populations with bacterial artificial chromosome constructs. J. Neurosci. 27, 9817–9823 10.1523/JNEUROSCI.2707-07.2007 17855595 PMC6672645

[B27] Graybiel A. M. (1984). Neurochemically specified subsystems in the basal ganglia. Ciba Found. Symp. 107, 114–149 6149896 10.1002/9780470720882.ch7

[B28] Graybiel A. M. (2004). Network-level neuroplasticity in cortico-basal ganglia pathways. Parkinsonism Relat. Disord. 10, 293–296 10.1016/j.parkreldis.2004.03.007 15196508

[B29] Graybiel A. M. Chesselet M. F. (1984). Compartmental distribution of striatal cell bodies expressing [Met]enkephalin-like immunoreactivity. Proc. Natl. Acad. Sci. U.S.A. 81, 7980–7984 10.1073/pnas.81.24.7980 6440146 PMC392277

[B30] Groh A. Meyer H. S. Schmidt E. F. Heintz N. Skamann B. Krieger P. (2010). Cell-type specific properties of pyramidal neurons in neocortex underlying a layout that is modifiable depending on the cortical area. Cereb. Cortex 20, 826–836 10.1093/cercor/bhp152 19643810

[B31] Haber S. N. Kim K. S. Mailly P. Calzavara R. (2006). Reward-related cortical inputs define a large striatal region in primates that interface with associative cortical connections, providing a substrate for incentive-based learning. J. Neurosci. 26, 8368–8376 10.1523/JNEUROSCI.0271-06.2006 16899732 PMC6673798

[B32] Hansson K. Jafari-Mamaghani M. Krieger P. (2013). RipleyGUI: software for analyzing spatial patterns in 3D cell distribution. Front. Neuroinform. 7:5 10.3389/fninf.2013.00005 23658544 PMC3620507

[B33] Hardman C. D. Henderson J. M. Finkelstein D. I. Horne M. K. Paxinos G. Halliday G. M. (2002). Comparison of the basal ganglia in rats, marmosets, macaques, baboons, and humans: volume and neuronal number for the output, internal relay, and striatal modulating nuclei. J. Comp. Neurol. 445, 238–255 10.1002/cne.10165 11920704

[B34] Heiman M. Schaefer A. Gong S. Peterson J. D. Day M. Ramsey K. E. (2008). A translational profiling approach for the molecular characterization of CNS cell types. Cell 135, 738–748 10.1016/j.cell.2008.10.028 19013281 PMC2696821

[B35] Herkenham M. Pert C. B. (1981). Mosaic distribution of opiate receptors, parafascicular projections and acetylcholinesterase in rat striatum. Nature 291, 415–418 10.1038/291415a0 6165892

[B36] Hervé D. Le Moine C. Corvol J. C. Belluscio L. Ledent C. Fienberg A. A. (2001). Galpha(olf) levels are regulated by receptor usage and control dopamine and adenosine action in the striatum. J. Neurosci. 21, 4390–4399 11404425 10.1523/JNEUROSCI.21-12-04390.2001 PMC6762749

[B37] Hippenmeyer S. Vrieseling E. Sigrist M. Portmann T. Laengle C. Ladle D. R. (2005). A developmental switch in the response of DRG neurons to ETS transcription factor signaling. PLoS Biol. 3:e159 10.1371/journal.pbio.0030159 15836427 PMC1084331

[B38] Jafari-Mamaghani M. Andersson M. Krieger P. (2010). Spatial point pattern analysis of neurons using Ripley's K-function in 3D. Front. Neuroinform. 4:9 10.3389/fninf.2010.00009 20577588 PMC2889688

[B39] Kawaguchi Y. (1993). Physiological, morphological, and histochemical characterization of three classes of interneurons in rat neostriatum. J. Neurosci. 13, 4908–4923 7693897 10.1523/JNEUROSCI.13-11-04908.1993 PMC6576359

[B40] Kawaguchi Y. (1997). Neostriatal cell subtypes and their functional roles. Neurosci. Res. 1, 1–8 10.1016/S0168-0102(96)01134-0 9089693

[B41] Kawaguchi Y. Wilson C. J. Emson P. C. (1990). Projection subtypes of rat neostriatal matrix cells revealed by intracellular injection of biocytin. J. Neurosci. 10, 3421–3438 1698947 10.1523/JNEUROSCI.10-10-03421.1990 PMC6570194

[B42] Koshimizu Y. Wu S. X. Unzai T. Hioki H. Sonomura T. Nakamura K. C. (2008). Paucity of enkephalin production in neostriatal striosomal neurons: analysis with preproenkephalin-green fluorescent protein transgenic mice. Eur. J. Neurosci. 28, 2053–2064 10.1111/j.1460-9568.2008.06502.x 19046386

[B43] Kravitz A. V. Freeze B. S. Parker P. R. Kay K. Thwin M. T. Deisseroth K. (2010). Regulation of parkinsonian motor behaviours by optogenetic control of basal ganglia circuitry. Nature 466, 622–626 10.1038/nature09159 20613723 PMC3552484

[B44] Kremer J. R. Mastronarde D. N. McIntosh J. R. (1996). Computer visualization of three-dimensional image data using IMOD. J. Struct. Biol. 116, 71–76 10.1006/jsbi.1996.0013 8742726

[B45] Lanca A. J. Boyd S. Kolb B. E. van der Kooy D. (1986). The development of a patchy organization of the rat striatum. Brain Res. 392, 1–10 3011213 10.1016/0165-3806(86)90226-9

[B46] Ledoux J. E. Farb C. R. Romanski L. M. (1991). Overlapping projections to the amygdala and striatum from auditory processing areas of the thalamus and cortex. Neurosci. Lett. 134, 139–144 10.1016/0304-3940(91)90526-Y 1815147

[B47] Lee K. S. Schubert P. Reddington M. Kreutzberg G. W. (1986). The distribution of adenosine A1 receptors and 5′-nucleotidase in the hippocampal formation of several mammalian species. J. Comp. Neurol. 246, 427–434 10.1002/cne.902460402 3009562

[B48] Lei W. Jiao Y. Del Mar N. Reiner A. (2004). Evidence for differential cortical input to direct pathway versus indirect pathway striatal projection neurons in rats. J. Neurosci. 24, 8289–8299 10.1523/JNEUROSCI.1990-04.2004 15385612 PMC6729697

[B49] Levesque M. Bedard A. Cossette M. Parent A. (2003). Novel aspects of the chemical anatomy of the striatum and its efferents projections. J. Chem. Neuroanat. 26, 271–281 10.1016/j.jchemneu.2003.07.001 14729129

[B50] Liu F. C. Graybiel A. M. (1992). Heterogeneous development of calbindin-D28K expression in the striatal matrix. J. Comp. Neurol. 320, 304–322 10.1002/cne.903200304 1351896

[B51] Mailly P. Haber S. N. Groenewegen H. J. Deniau J. M. (2009). A 3D multi-modal and multi-dimensional digital brain model as a framework for data sharing. J. Neurosci. Methods 194, 56–63 10.1016/j.jneumeth.2009.12.014 20043949

[B52] Mailly P. Haber S. N. Groenewegen H. J. Deniau J. M. (2010). A 3D multi-modal and multi-dimensional digital brain model as a framework for data sharing. J. Neurosci. Methods 194, 56–63 10.1016/j.jneumeth.2009.12.014 20043949

[B53] Matamales M. Bertran-Gonzalez J. Salomon L. Degos B. Deniau J. M. Valjent E. (2009). Striatal medium-sized spiny neurons: identification by nuclear staining and study of neuronal subpopulations in BAC transgenic mice. PLoS ONE 4:e4770 10.1371/journal.pone.0004770 19274089 PMC2651623

[B54] Miyoshi G. Hjerling-Leffler J. Karayannis T. Sousa V. H. Butt S. J. Battiste J. (2010). Genetic fate mapping reveals that the caudal ganglionic eminence produces a large and diverse population of superficial cortical interneurons. J. Neurosci. 30, 1582–1594 10.1523/JNEUROSCI.4515-09.2010 20130169 PMC2826846

[B55] Nicola S. M. (2007). The nucleus accumbens as part of a basal ganglia action selection circuit. Psychopharmacology (Berl.) 191, 521–550 10.1007/s00213-006-0510-4 16983543

[B56] Reiner A. Hart N. M. Lei W. Deng Y. (2010). Corticostriatal projection neurons - dichotomous types and dichotomous functions. Front. Neuroanat. 4:142 10.3389/fnana.2010.00142 21088706 PMC2982718

[B57] Reiner A. Jiao Y. Del Mar N. Laverghetta A. V. Lei W. L. (2003). Differential morphology of pyramidal tract-type and intratelencephalically projecting-type corticostriatal neurons and their intrastriatal terminals in rats. J. Comp. Neurol. 457, 420–440 10.1002/cne.10541 12561080

[B58] Ripley B. D. (1988). Statistical Interference for Spatial Processes. Cambridge: Cambridge University Press 10.1017/CBO9780511624131

[B59] Sako W. Morigaki R. Nagahiro S. Kaji R. Goto S. (2010). Olfactory type G-protein a subunit in striosome-matrix dopamine systems in adult mice. Neuroscience 170, 497–502 10.1016/j.neuroscience.2010.06.072 20603191

[B60] Schoen S. W. Graybiel A. M. (1993). Species-specific patterns of glycoprotein expression in the developing rodent caudoputamen: association of 5′-nucleotidase activity with dopamine islands and striosomes in rat, but with extrastriosomal matrix in mouse. J. Comp. Neurol. 333, 578–596 10.1002/cne.903330410 8103780

[B61] Selemon L. D. Goldman-Rakic P. S. (1985). Longitudinal topography and interdigitation of corticostriatal projections in the rhesus monkey. J. Neurosci. 5, 776–794 2983048 10.1523/JNEUROSCI.05-03-00776.1985 PMC6565017

[B62] Shepherd G. M. (2013). Corticostriatal connectivity and its role in disease. Nat. Rev. Neurosci. 14, 278–291 10.1038/nrn3469 23511908 PMC4096337

[B63] Shu S. Y. Bao X. Li S. Niu D. Xu Z. Li Y. (1999). A new subdivision of mammalian neostriatum with functional implications to learning and memory. J. Neurosci. Res. 58, 242–253 10502280

[B64] Shu S. Y. McGinty J. F. Peterson G. M. (1990). High density of zinc-containing and dynorphin B- and substance P-immunoreactive terminals in the marginal division of the rat striatum. Brain Res. Bull. 24, 201–205 10.1016/0361-9230(90)90206-F 1691046

[B65] Shu S. Y. Penny G. R. Peterson G. M. (1988). The ‘marginal division’: a new subdivision in the neostriatum of the rat. J. Chem. Neuroanat. 1, 147–163 2477034

[B66] Srinivas S. Watanabe T. Lin C. S. William C. M. Tanabe Y. Jessell T. M. (2001). Cre reporter strains produced by targeted insertion of EYFP and ECFP into the ROSA26 locus. BMC Dev. Biol. 1:4 10.1186/1471-213X-1-4 11299042 PMC31338

[B67] Tepper J. M. Tecuapetla F. Koos T. Ibanez-Sandoval O. (2010). Heterogeneity and diversity of striatal GABAergic interneurons. Front. Neuroanat. 4:150 10.3389/fnana.2010.00150 21228905 PMC3016690

[B68] Valjent E. Bertran-Gonzalez J. Hervé D. Fisone G. Girault J. A. (2009). Looking BAC at striatal signaling: cell-specific analysis in new transgenic mice. Trends Neurosci. 32, 538–547 10.1016/j.tins.2009.06.005 19765834

[B69] Wu Y. Richard S. Parent A. (2000). The organization of the striatal output system: a single-cell juxtacellular labeling study in the rat. Neurosci. Res. 38, 49–62 10.1016/S0168-0102(00)00140-1 10997578

